# Perceived current and future roles of UK-based community pharmacy professionals in the long-term management of acne

**DOI:** 10.1016/j.rcsop.2023.100310

**Published:** 2023-07-20

**Authors:** Kimberley Sonnex, Tracey Thornley, Naomi Fleming, Alishah Lakha, Donna M. Lecky, Indira Pillay, Shazia Patel, Claire Anderson, Matthew Boyd, Diane Ashiru-Oredope

**Affiliations:** a University of Nottingham, Nottingham, UK; b UK Health Security Agency, UK; c NHS England, UK; d Derbyshire Integrated Care Board, UK

**Keywords:** Acne, Antimicrobial stewardship, Antimicrobial resistance, Community pharmacy, Pharmacist, Pharmacy technician

## Abstract

**Introduction:**

Acne is a common skin condition treated in community pharmacy but moderate to severe cases may need referral to general practice for treatment that may include topical or oral antimicrobial treatments. Pharmacy teams working in the community are well-placed to manage acne treatments in line with NICE guidance.

**Objectives:**

To explore the perceived current and future roles of community pharmacy (CP) teams alongside needs to achieve potential future roles. Additionally, usefulness of the TARGET acne ‘How to’ toolkit to support these roles was sought.

**Methods:**

A mixed-methods electronic survey of UK-based CP professionals and stakeholders in March 2023.

**Results:**

54 pharmacy professionals and stakeholders responded to the survey. The current confidence of pharmacy professionals in managing acne was rated as moderate and reviewing long-term medications for acne prescribed by another healthcare professional was seen as a future role. The needs identified to undertake such a role were: training, availability of prescribing or supply frameworks, and appropriate remuneration. The TARGET acne resources were thought of as being very useful for acne management.

**Conclusions:**

Potential future roles for CP have been identified, alongside additional needs to undertake these roles; the TARGET acne ‘How to’ resources could support pharmacy professionals in the management and review of antimicrobial treatment for acne.

## 1 Introduction

Acne (acne vulgaris) is a common skin condition affecting up to 85% of 12 to 24 year-olds.^1^ Mild cases can be managed effectively by community pharmacies, with topical treatments available over the counter and skin care advice,^2^ and as part of the NHS Minor Ailments Scheme, which allowed people who don't pay prescription charges to access medicines for minor ailments (including acne) free of charge from a community pharmacy.^3^, ^4^, ^5^

Moderate and severe cases of acne may need referral to general practice for other treatments to be prescribed, including topical or oral antibiotic therapy^6^ and a recent study showed that 44.5% of people with a new acne diagnosis received a prescription for long-term oral antibiotics.^7^ Available guidance on the management of acne states that only exceptional circumstances should lead to use of antibiotics for over six months^8^; long-term use of topical and oral antibiotics is associated with antimicrobial resistance (AMR). As general practice generates over 72% of total antibiotic prescriptions in England,^9^ ongoing and regular patient reviews by healthcare professionals are vital to support antimicrobial prescribing appropriateness and ensure alignment with NICE guidance the NHS long-term plan.^10^

Therefore, community pharmacy teams have a dual role in the management of acne; supply of over the counter medication for mild cases and supply and review of long-term medications, including antibiotics for moderate to severe cases. However, little is known about the perceptions and confidence in delivery of these roles in community pharmacy.

Additionally, with the increased role and funding of independent prescribing status in UK community pharmacy,^11^ there is the opportunity for community pharmacy professionals to be further involved in the long-term management of acne. However, there is mixed evidence on the clinical appropriateness of community pharmacies in the diagnosis and management of acne,^12^^,^^13^ highlighting the possible need for further training.

With this in mind, the UK Health Security Agency (UKHSA) led TARGET (Treat Antibiotics Responsibly, Guidance, Education and Tools) antibiotics toolkit,^14^ was designed to support primary care clinicians to champion and implement antimicrobial stewardship (AMS) activities, and is hosted on the Royal College of General Practitioners (RCGP) website. An evidence-based intervention (‘How to…’ tool) to review long-term or repeat antimicrobial prescriptions for acne was developed with a multidisciplinary group. Other AMR resources have previously been successfully embedded in the community pharmacy contractual framework.^15^

The aim of this study was to describe the perceived current and future roles of community pharmacy professionals (pharmacists and pharmacy technicians) in the management of acne, including activities related to antimicrobial stewardship (AMS), and the additional capability, opportunity and motivational needs required to meet this role. Subsequently, the potential usefulness of the TARGET acne ‘How to…’ resources in supporting the previously identified roles of community pharmacy professionals was assessed.

## 2 Materials and methods

### 2.1 Study design and participants

A mixed-methods, but primarily quantitative study was undertaken. Participants were community pharmacy professionals based in the UK and community pharmacy stakeholders. The stakeholders, such as Local Pharmaceutical Committee (LPC) leads, professional services users (e.g. General Practitioners) and professional leads (e.g. Royal Pharmaceutical Society) were surveyed to understand their views on perceived current and future roles of CP teams in the management of acne, with insights into any contractual and operational frameworks that may influence this.

### 2.2 Survey method

Two electronic questionnaires were developed where participants rated their agreement with predefined statements. The surveys were deployed using the Qualtrics XM platform. The current and future roles of community pharmacy professionals were assessed via ‘yes/no’ responses to 11 questions on areas of practice. The COM-B model^16^ was used to assess the capability, opportunity and motivational needs of pharmacists and pharmacy technicians working in community pharmacy around acne management by rating their agreement with predefined statements on a 5-point Likert scale.

The community pharmacy survey was distributed widely via pharmacy networks such as LPCs, the Company Chemists' Association, and advertised on social media. The stakeholder survey was also sent to key community pharmacy stakeholders; such as pharmacy representatives (LPC leads and community pharmacy education leads), professional service users (community pharmacy integration leads), and professional bodies (including the Royal Pharmaceutical Society and Royal College of General Practitioners). This was done through contacts of the survey project team.

Both surveys were piloted for the first few days of the study and amended as needed. The final surveys used can be found in the supplementary material. Each community pharmacy participant who agreed to it was entered into a draw for one of 20 £25 Amazon vouchers. No remuneration was given to stakeholders completing the survey as it was assumed they would respond in the course of their usual work. Previous research has shown low engagement from community pharmacists in research, citing lack of remuneration as a barrier^17^; so a small financial incentive was given to support increased uptake.

The TARGET acne ‘How to…’ toolkit and clinical scenarios can be accessed via the RCGP TARGET antibiotics toolkit hub^14^.

### 2.3 Data analysis

Question types included 5-point Likert questions (scale 1 to 5; strongly disagree to strongly agree), yes/no and free-text response. Mean and standard deviation of the 5-point Likert responses and percentage of yes/no responses were calculated. Demographic data on the profession of the participants, duration of professional registration and region of the UK they practiced in were also collected.

### 2.4 Ethical approval

This study was reviewed and approved by the University of Nottingham School of Pharmacy Research Ethics Committee (Ref: 009–2023).

## 3 Results

A total of 44 pharmacy professionals responded to at least one question in the community pharmacy survey, with 36 completing fully. There were 90 survey initiations (clicks on the survey link); a completion rate of 40%. Pharmacists made up most of respondents: with 37 responses, 2 were pharmacy technicians and 5 ‘other’ (who were undefined but may include pharmacy assistants or managers). When asked about AMS training 11 out of 44 (25%) respondents stated they had not done any previous training.

Responses were received from participants from England, Wales and Northern Ireland, and with a range of post-registration experience. Ten stakeholders responded to the stakeholder survey. Full demographic data can be found in the supplementary material.

### 3.1 Roles of community pharmacy in managing acne

Areas of acne management that rated low currently, but much higher as a future role were referral to secondary healthcare professionals, prescribing, and review of medications prescribed by someone else (Table 1). The results are presented as pooled data for both the community pharmacy professionals and stakeholders; initially it had been hoped that a comparison in responses could be made between the two groups, however low number of responses meant this was not statistically meaningful.

**Table 1:** Current and future roles of community pharmacy professionals in acne management (pooled data from community pharmacy and stakeholder respondents, *n* = 54).

	‘Yes’ respondents (%)	
	Current	Future^1^
Over the counter advice	50 (92.6)	28 (51.9)
Supply of over-the-counter products	52 (96.3)	30 (55.6)
Skin cleansing regime and self-care advice	48 (88.9)	26 (48.1)
Supply of long-term medications prescribed by someone else	48 (88.9)	27 (50.0)
Review of long-term medications prescribed by someone else	17 (31.5)	44 (81.5)
Referral to general practice	48 (88.9)	24 (44.4)
Referral to dermatology specialist	5 (9.3)	50 (92.6)
Prescribing of topical antibiotics	12 (22.2)	48 (88.9)
Prescribing of oral antibiotics	6 (11.1)	48 (88.9)

1 Question may have been interpreted as ‘what is a new role for community pharmacy professionals’.

### 3.2 Capability, opportunity and motivational needs in managing acne

Community pharmacy professionals rated their current capability in managing acne as 3.75 (S.D. 1.08) on a 5-point Likert scale (Table 2). The most important factors to allow pharmacy professionals to undertake future extended roles in acne management in terms of opportunity and motivation were protected training time, availability of patient group directions for treatment (PGD, a legal framework to allow certain healthcare professionals to supply specified medicines without a prescription) and appropriate remuneration.

**Table 2:** 5-point Likert scale responses to capability, opportunity and motivation needs to undertake extended roles in acne management as answered by community pharmacy professionals (*n* = 36).

Capability	
	Mean (S.D.)
I am confident in my current role in managing acne	3.75 (1.07)
Opportunity need	
Protected time for education and training	4.21 (0.88)
Shadowing opportunity where this is already in place	3.55 (0.92)
Community of practice to share experiences	3.64 (0.98)
Addition of acne to Community Pharmacy Consultation Service^1^	3.97 (1.00)
Local Enhanced Service	4.00 (1.07)
Patient Group Directions for topical and oral acne treatments	4.30 (0.87)
Prescribing qualification	3.79 (1.07)
Joint working with General Practice (GP) or Primary Care Network (PCN)	3.55 (1.05)
Read/write access to medical records	4.18 (0.83)
Addition to pharmacy antimicrobial stewardship action plan	3.88 (1.02)
Access to referral pathways such as dermatology or mental health services	4.18 (0.83)
Motivation need	
Addition of acne to Community Pharmacy Consultation Service (CPCS)^1^	4.24 (1.06)
Local Enhanced Service	4.18 (1.10)
Addition to pharmacy antimicrobial stewardship action plan	3.91 (1.09)
Improved quality of patient care	4.42 (0.82)
Improved access to treatment for patient	4.52 (0.86)
Job satisfaction	4.30 (0.90)
Contributing to reduction in anti-microbial resistance	4.21 (1.04)
Appropriate remuneration	4.64 (0.59)
Improved recognition of community pharmacy skills by GPs and/or PCN	4.48 (0.50)
Improved recognition of community pharmacy skills by patients	4.64 (0.77)

1 Community Pharmacy Consultation Service (CPCS) is an NHS funded service as part of the pharmacy contractual framework.^15^

### 3.3 Use of the TARGET ‘How to…’ resources to manage acne

When asked if they were already familiar with the TARGET toolkit resources before the survey 27 (50%) of both stakeholders and community pharmacy professionals responded ‘yes’ but only 3 (7%) had seen the specific acne toolkit.

Community pharmacy professionals were asked about their agreement with 5-point Likert questions on the usefulness of the acne resources in their current and potential future role of managing acne (Table 3). There was strong agreement that the TARGET acne resources were useful in a future role (5-point Likert score: 4.32, SD 0.16) and somewhat agreement that they were useful in their current role (5-point Likert score: 3.79, SD 0.45).

**Table 3:** Usefulness of the TARGET ‘How to…’ resources in the current and future roles of in managing acne rated on a 5-point Likert scale (1 = not at all useful to 5 = extremely useful) as answered by community pharmacy professionals (*n* = 17).

The ‘how to’ resource supports me to do the following in my role:	Mean (S.D.)	
	Current role	Future role
Diagnose acne vulgaris	4.12 (0.83)	4.29 (0.82)
Provide self-care advice	4.59 (0.49)	4.59 (0.49)
Initiate topical treatment for mild or moderate acne	4.29 (0.67)	4.35 (0.68)
Initiate topical and oral treatment for moderate/severe acne	3.59 (1.14)	4.41 (0.77)
Make referrals to a general practice as required	4.24 (0.81)	4.41 (0.77)
Make referrals to a dermatologist as required	3.41 (1.14)	4.35 (0.90)
Make referrals to mental health services as required	3.65 (1.08)	4.12 (1.13)
Review current treatment and swap topical treatments for acne	3.53 (1.19)	4.24 (0.94)
Review current treatment of acne and trial off antibiotics	3.35 (1.28)	4.24 (0.94)
Review current treatment and add in oral antibiotics	3.18 (1.38)	4.18 (0.93)
*Mean score*	*3.79 (0.45)*	*4.32 (0.16)*

Both stakeholder and community pharmacy professionals were asked about specific parts of the acne resources that were thought to be most useful (Table 4). The sections on information on acne, risk factors, self-care, treatment and the clinical scenarios were the most useful, however all sections were deemed at least somewhat useful (5-point Likert score: 4.22; SD 0.24).

**Table 4:** Usefulness rated on 5-point Likert scale (1 = not at all useful to 5 = extremely useful) of sections of the TARGET acne ‘How to’ resources as answered by community pharmacy professionals and stakeholders (*n* = 25).

	Mean (S.D.)
Information on Acne	4.48 (0.64)
Information on aggravating and modifiable risk factors	4.44 (0.64)
Undertake baseline search and analysis	4.12 (0.91)
Develop implementation plan	4.16 (0.92)
During the patient consultation	4.36 (0.74)
Self-care measures	4.44 (0.50)
Treatment of acne vulgaris	4.40 (0.57)
Referral to specialist care	3.92 (1.06)
Flowchart to review long-term and repeated antibiotic use in acne	4.32 (0.73)
Undertake post review search and analysis	3.76 (1.14)
Share key themes and embed quality improvement practice	3.88 (1.07)
Acne clinical scenarios	4.40 (0.85)
*Mean score*	*4.22 (0.24)*

## 4 Discussion

This study has described the current and perceived potential roles of community pharmacy teams in the management of acne, including AMS, and the additional capability, opportunity and motivational needs required to deliver this role. The usefulness of the acne resources in supporting the current and future roles of community pharmacy was also explored.

### 4.1 Roles of community pharmacy in managing acne

A high percentage (89–96%) of respondents said that over the counter advice and supply of medications (both prescription and non-prescription) were current roles of community pharmacy in managing acne, traditional roles that fit with the available guidance.^2^^,^^6^ However, when asked if these activities were a future role, the response was much lower (50–56%); possibly indicating an evolving role for community pharmacy teams; supported by the interim NHS people plan^18^ to devolve more responsibility to pharmacy technicians. Reviewing of long-term medications prescribed by someone else was only thought of as being a current role by 32% of respondents but a future role by 82%; possibly indicating that it is seen as an extended prescribing role. Reviewing of long-term medications for acne, including antibiotics, is essential to good AMS as per the national guidance on acne management.^8^

### 4.2 Capability, opportunity and motivation

Community pharmacy professionals rated their current confidence in managing acne as moderate. A high percentage had not undertaken any pharmacy specific AMS training, indicating a training need in this area. Questions on factors that are important for the capability, opportunity and motivation of community pharmacy professionals highlighted remuneration and training as the most common needs. Additionally, arrangements such as supply of medicines via PGDs were also thought to be necessary and more highly rated by community pharmacy professionals than an independent prescribing qualification, despite the current funding available for such a qualification.^11^ Time-pressures on community pharmacy professionals and lack of interest in achieving this qualification may be influential in this response; a recent systematic review has demonstrated that barriers to independent prescribing qualification in primary care are multifactorial.^19^ Other highly scoring motivators included improved recognition of community pharmacy skills by patients and GPs and improved access to treatment for patient.

Pharmacy services in England are funded as per the Community Pharmacy Contractual Framework,^15^ which makes provision for new and expanded services such as the Community Pharmacy Consultation Service (CPCS). Addition of an extended role in managing acne to CPCS was rated highly as a motivational need by community pharmacy professionals (5-point Likert scale: 4.24, SD 1.06), possibly due to this service being recognised and successful.^20^

### 4.3 The TARGET acne ‘How to’ resources

The acne resources were rated more highly to support community pharmacy staff in extended future roles than in their current roles. Some parts of the resource were deemed more useful for community pharmacy professionals than others (Table 4) and this provides a starting point for the resource to be adapted to training needs identified by community pharmacy teams to improve confidence in acne consultations.

Community pharmacy professionals may benefit from having access to the resources to support patients with acne, particularly those on long term treatment with antibiotic therapy. This builds on the successful implementation of the TARGET antibiotic checklist in the Pharmacy Quality Scheme (PQS) in England.^15^

### 4.4 Limitations

There are two main limitations of this study; firstly, low response rate and secondly lack of follow-up to gain further insight into the survey responses. Only 44 responses were received from community pharmacy professionals despite the survey being circulated widely via pharmacy networks and social media. This may indicate that work pressures have limited community pharmacy involvement in research-type activities, which has been demonstrated previously.^17^ Furthermore, as a financial incentive was offered to participants this may have influenced the people who chose to respond and with only 44 respondents, it is not possible to apply the findings more widely to the profession. As this was a pilot study there is the opportunity to adapt the TARGET ‘How to’ resources for community pharmacy and subsequently further explore the roles, capability, motivation and opportunity of community pharmacy professionals in managing acne.

## 5 Conclusions

Despite the management of mild acne being well established in community pharmacy, the pharmacy professionals surveyed do not see the review of long-term medications for acne as part of their current role.

The TARGET acne ‘How to’ resources are thought to be useful in both the current and future roles of community pharmacy to manage acne but may need adaptation to be more specific to community pharmacy teams. Furthermore, there is the potential to use these resources to upskill pharmacy professionals in the review of long-term medications for acne, which was perceived as a future role for community pharmacy teams.

## Funding

UKHSA.

All authors declare that there's no financial/personal interest or belief that could affect their objectivity in the submission of this manuscript

## Declaration of Competing Interest

None.

## Appendix A Supplementary data

Supplementary material 1: Community pharmacy and stakeholder surveys Image 1

Supplementary material 2: Demographic data Image 2
