# Supplementary material for: Perceived current and future roles of UK-based community pharmacy professionals in the long-term management of acne
Source: Explor Res Clin Soc Pharm. 2023 Jul 20;11:100310. doi: 10.1016/j.rcsop.2023.100310 (PMC10460989; doi:10.1016/j.rcsop.2023.100310)
Supplement: Supplementary file 1 — Supplementary material 1: Community pharmacy and stakeholder surveys [file mmc1.pdf]

In collaboration with UKHSA and NHS England AMR programme teams

Introduction

Acne Management Survey

Community pharmacies are an integral part of the NHS and often the first port of call for minor ailments, healthy living support as well as skin conditions such as acne. UKHSA and NHS E AMR teams are working collaboratively with University of Nottingham to evaluate the "acne how to" resources within primary care, as well as understanding how this tool and associated resources could be used to provide additional support on acne management for community pharmacy teams. This includes reviewing of patients on repeated treatments for acne in order to make recommendations to prescribers and enabling community pharmacists to supply acne treatments according to available guidelines or protocols.

As a key stakeholder we would appreciate you spending 5-10 minutes to complete the survey below to help us identify the current roles of community pharmacy in acne management and what support resources may be useful. Please read the participant information sheet (link below) before completing. Whilst not the main focus of this survey, any additional feedback that you may have on the suitability of current acne 'How to' resources (links below) would also be appreciated.

Please submit your completed survey by **5pm 21st March**

By continuing with this survey you are consenting to us recording your responses

We would also appreciate you circulating the survey we are sending to community pharmacy teams in your networks. The link for this survey is here: Community pharmacy acne survey

Links to the acne 'How to' resource and clinical scenarios<br>TARGET acne 'How to' resource<br>Acne clinical scenarios<br><br>If you would like further information about this survey, please read the participant information sheet below:<br>Participant Information Sheet

Block 3

Please select the group you are responding from:

- ○ Pharmacy representative
- ○ Professional body
- ○ NHS organisation
- ○ NHS other

Please select the area that you represent

- ○ National
- ○ Regional or local area

Block 1

Which of the following do you see as the .... (select ALL that apply)

	**current** role of community pharmacy in managing patients with acne?	**future** role of community pharmacy in managing patients with acne?
Over the counter advice	☐	☐
Supply of over the counter products	☐	☐
Skin cleansing regime and self-care advice	☐	☐
Supply of long-term medications prescribed by someone else	☐	☐
Review of long-term medications prescribed by someone else	☐	☐
Referral to general practice	☐	☐

	**current** role of community pharmacy in managing patients with acne?	**future** role of community pharmacy in managing patients with acne?
Referral to dermatology specialist	☐	☐
Prescribing of topical antibiotics	☐	☐
Prescribing of oral antibiotics	☐	☐
No role	☐	☐
Other role (please state below) ______	☐	☐

What further support is required for community pharmacy to be able to manage long-term medications for patients with acne (by supply, review and/or prescribing)? Select ALL that apply

- ☐ Independent prescribing qualification
- ☐ Shadowing opportunities
- ☐ Webinars
- ☐ Distance learning resources
- ☐ Community of practice to share experiences
- ☐ No further training needed
- ☐ Other (please state) ______

What would provide community pharmacy with the **opportunity** to undertake management of long-term medications for patients with acne (by supply, review and/or prescribing)? Select ALL that apply

- ☐ Addition of acne to CPCS
- ☐ Local Enhanced Service
- ☐ PGDs for topical and oral acne treatments
- ☐ Access to referral pathways
- ☐ Joint working with GP practice or PCN
- ☐ Read/write access to medical records
- ☐ No further opportunity needed
- ☐ Other (please state) ______

What could be done to **motivate** community pharmacy to undertake management of long-term medications for patients with acne (by supply, review and/or prescribing)? Select ALL that apply

- ☐ Addition of acne to CPCS
- ☐ Local Enhanced Service
- ☐ Addition to pharmacy antimicrobial stewardship action plan
- ☐ No further motivation needed
- ☐ ______ Other (please state)

Were you aware of the TARGET toolkit resources prior to this survey?

- ○ Yes

- No

Had you seen the Acne 'How to….' resource prior to this survey?

- Yes
- No

[TARGET acne 'How to' resource]

[Acne clinical scenarios]

How likely do you feel these resources would upskill community pharmacy to initiate over the counter acne treatment?

- Extremely unlikely
- Somewhat unlikely
- Neither likely nor unlikely

Somewhat likely

○

Extremely likely

○

How likely do you feel these resources would upskill community pharmacists to review prescribed acne treatment?

Extremely unlikely

○

Somewhat unlikely

○

Neither likely nor unlikely

○

Somewhat likely

○

Extremely likely

○

The acne 'How to' resource has been developed to support pharmacists in GP practices review patients with acne.

However, if this resource were to be further developed for use in community pharmacy, please rate the usefulness of each of the following sections to community pharmacy (available here: Acne 'How to' and Acne clinical scenarios)

	Strongly disagree	Somewhat disagree	Neither agree nor disagree	Somewhat agree	Strongly agree
2.1 Information on Acne	○	○	○	○	○
2.2. Information on aggravating and modifiable risk factors	○	○	○	○	○
3.1 Step 1: Undertake baseline search and analysis	○	○	○	○	○
3.2 Step 2: Develop implementation plan	○	○	○	○	○
3.3.1 During the patient consultation	○	○	○	○	○
3.3.1.1 Self-care measures	○	○	○	○	○
3.3.2 Treatment of acne vulgaris	○	○	○	○	○

	Strongly disagree	Somewhat disagree	Neither agree nor disagree	Somewhat agree	Strongly agree
3.3.3 Referral to specialist care	○	○	○	○	○
3.3.4 Flowchart to review long-term and repeated antibiotic use in acne	○	○	○	○	○
3.4 Step 4: Undertake post review search and analysis	○	○	○	○	○
3.5 Step 5: Share key themes and embed quality improvement practice	○	○	○	○	○
Acne clinical scenarios	○	○	○	○	○

Please give us any further feedback you have on the ‘How to’ resource

Please give us any further feedback you have on the acne case study PowerPoint

Community of Practice

Would you be interested in joining a community of practice for antimicrobial stewardship?

- ○ Yes
- ○ No

Please provide your details below so that we have permission to contact you. Your details will not be linked to your survey responses

Name

Email address

Powered by Qualtrics
