# Supplementary material for: Perceived current and future roles of UK-based community pharmacy professionals in the long-term management of acne
Source: Explor Res Clin Soc Pharm. 2023 Jul 20;11:100310. doi: 10.1016/j.rcsop.2023.100310 (PMC10460989; doi:10.1016/j.rcsop.2023.100310)
Supplement: Supplementary file 2 — Supplementary material 2: Demographic data [file mmc2.pdf]

In collaboration with UKHSA and NHS England AMR programme teams

Introduction

Acne Management Survey

Community pharmacies are an integral part of the NHS and often the first port of call for minor ailments, healthy living support as well as skin conditions such as acne. UKHSA and NHS E AMR teams are working collaboratively with University of Nottingham to evaluate the "acne how to" resources within primary care, as well as understanding how this tool and associated resources can provide additional support on acne management for community pharmacy teams. This includes reviewing of patients on repeated treatments for acne in order to make recommendations to prescribers, and enabling community pharmacy to supply acne treatments according to available guidelines or protocols.

As a UK registered pharmacist or pharmacy technician practising in the community, we would appreciate you spending 5-10 minutes to complete the survey below to help us identify your current roles in acne management and what support resources you would find useful. Please read the participant information sheet (link below) before you start. In addition, you will be able to access the acne 'How to' resource to undertake a CPD cycle. If you do decide to undertake this learning and

provide supplementary feedback, then you will be offered the opportunity to be entered into a prize draw to win one of 20 x £25 amazon vouchers

Please submit your completed questionnaire by **5pm 21st March**

By continuing with this survey you are consenting to us recording your responses

Links to the acne 'How to' resource and clinical scenarios<br>TARGET acne 'How to' resource<br>Acne clinical scenarios<br>If you would like further information about this survey, please read the participant information sheet below:<br>Participant Information Sheet

Block 4

Please click below to confirm this is a genuine response

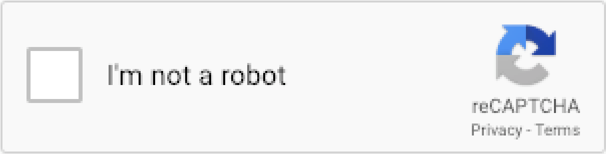

Block 1

Please select which of the following you are responding as:

- ○ UK registered pharmacist
- ○ UK registered pharmacy technician
- ○ Other

Have you done any training on antimicrobial stewardship before?

Yes	No
○	○

Please tell us about the training you have done

[ ]

Were you aware of the TARGET toolkit resources prior to this survey?

Yes	No
○	○

Had you seen the Acne 'How to….' resource before this survey?

Yes	No
○	○

Block 2

Which of the following do you see as the .... (select ALL that apply)

	**current** role of community pharmacy in managing patients with acne?	**future** role of community pharmacy in managing patients with acne?
Over the counter advice	☐	☐
Supply of over the counter products	☐	☐
Skin cleansing regime and self-care advice	☐	☐
Supply of long-term medications prescribed by someone else	☐	☐
Review of long-term medications prescribed by someone else	☐	☐
Referral to general practice	☐	☐

	**current** role of community pharmacy in managing patients with acne?	**future** role of community pharmacy in managing patients with acne?
Referral to dermatology specialist	☐	☐
Prescribing of topical antibiotics	☐	☐
Prescribing of oral antibiotics	☐	☐
No role	☐	☐
Other role (please state below) ____	☐	☐

Please indicate to what extent you agree with the following statement:

I am confident in my current role in managing acne

Strongly disagree ○

Somewhat disagree ○

Neither agree nor disagree ○

Somewhat agree

○

Strongly agree

○

[TARGET acne 'How to' resource]

[Acne clinical scenarios]

Have you accessed the acne ‘how to’ resource and case studies attached to this survey?

○ Yes

○ No

Having accessed the acne ‘how to’ resource...

To what extent you agree with the following statements

The 'how to' resource supports me, or will support me, to do the following in my **current** role:

	Strongly disagree	Somewhat disagree	Neither agree nor disagree	Somewhat agree	Strongly agree
Diagnose acne vulgaris	○	○	○	○	○

	Strongly disagree	Somewhat disagree	Neither agree nor disagree	Somewhat agree	Strongly agree
Provide self-care advice	○	○	○	○	○
initiate topical treatment for mild or moderate acne	○	○	○	○	○
Initiate topical and oral treatment for moderate/severe acne	○	○	○	○	○
Make referrals to general practice as required	○	○	○	○	○
Make referrals to a dermatologist as required	○	○	○	○	○
Make referrals to mental health services as required	○	○	○	○	○
Review current treatment and swap topical treatments for acne	○	○	○	○	○
Review current treatment of acne and trial off antibiotics	○	○	○	○	○

	Strongly disagree	Somewhat disagree	Neither agree nor disagree	Somewhat agree	Strongly agree
Review current treatment and add in oral antibiotics	○	○	○	○	○

The 'how to' resource supports me to do the following in the **future**, if there were to be an extended role for community pharmacy

	Strongly disagree	Somewhat disagree	Neither agree nor disagree	Somewhat agree	Strongly agree
Diagnose acne vulgaris	○	○	○	○	○
Provide self-care advice	○	○	○	○	○
initiate topical treatment for mild or moderate acne	○	○	○	○	○
Initiate topical and oral treatment for moderate/severe acne	○	○	○	○	○
Make referrals to general practice as required	○	○	○	○	○

	Strongly disagree	Somewhat disagree	Neither agree nor disagree	Somewhat agree	Strongly agree
Make referrals to a dermatologist as required	○	○	○	○	○
Make referrals to mental health services as required	○	○	○	○	○
Review current treatment and swap topical treatments for acne	○	○	○	○	○
Review current treatment of acne and trial off antibiotics	○	○	○	○	○
Review current treatment and add in oral antibiotics	○	○	○	○	○

The acne 'How to' resource has been developed to support pharmacists in GP practices review patients with acne.

However, if this resource were to be further developed for use in community pharmacy, please rate the usefulness of each of the following sections to community pharmacy (available here: Acne 'How to' and Acne clinical scenarios)

	Strongly disagree	Somewhat disagree	Neither agree nor disagree	Somewhat agree	Strongly agree
2.1 Information on Acne					
2.2. Information on aggravating and modifiable risk factors					
3.1 Step 1: Undertake baseline search and analysis					
3.2 Step 2: Develop implementation plan					
3.3.1 During the patient consultation					
3.3.1.1 Self-care measures					
3.3.2 Treatment of acne vulgaris					
3.3.3 Referral to specialist care					
3.3.4 Flowchart to review long-term and repeated antibiotic use in acne					
3.4 Step 4: Undertake post review search and analysis					

	Strongly disagree	Somewhat disagree	Neither agree nor disagree	Somewhat agree	Strongly agree
3.5 Step 5: Share key themes and embed quality improvement practice	○	○	○	○	○
Acne clinical scenarios	○	○	○	○	○

Please use this section to provide any additional feedback on the acne ‘How to’ resource to support the community pharmacy in an extended role in acne management

Please use this section to provide any additional feedback on the clinical scenarios to support the community pharmacy in an extended role in acne management

How important are each of the following options to provide you with the **opportunity** to undertake extended roles in acne management in community pharmacy

	Not at all important	Slightly important	Moderately important	Very important	Extremely important
Protected time for education and training	◯	◯	◯	◯	◯
Shadowing opportunity where this is already in place	◯	◯	◯	◯	◯
Community of practice to share experiences	◯	◯	◯	◯	◯
Addition of acne to CPCS	◯	◯	◯	◯	◯
Local Enhanced Service	◯	◯	◯	◯	◯
PGDs for topical and oral acne treatments	◯	◯	◯	◯	◯
Prescribing qualification	◯	◯	◯	◯	◯
Joint working with GP practice or PCN	◯	◯	◯	◯	◯
Read/write access to medical records	◯	◯	◯	◯	◯
Addition to pharmacy antimicrobial stewardship action plan	◯	◯	◯	◯	◯
Access to referral pathways such as dermatology or mental health services	◯	◯	◯	◯	◯

	Not at all important	Slightly important	Moderately important	Very important	Extremely important
Other (please state) [ ]	○	○	○	○	○

To what extent would each of the following options **motivate** you to undertake extended roles in acne management in community pharmacy

	Extremely unlikely	Somewhat unlikely	Neither likely nor unlikely	Somewhat likely	Extremely likely
Addition of acne to CPCS	○	○	○	○	○
Local Enhanced Service	○	○	○	○	○
Addition to pharmacy antimicrobial stewardship action plan	○	○	○	○	○
Improved quality of patient care	○	○	○	○	○
Improved access to treatment for patient	○	○	○	○	○
Job satisfaction	○	○	○	○	○
Contributing to reduction in antimicobial resistance	○	○	○	○	○
Appropriate remuneration	○	○	○	○	○

	Extremely unlikely	Somewhat unlikely	Neither likely nor unlikely	Somewhat likely	Extremely likely
Improved recognition of community pharmacy skills by GPs and/or PCN	○	○	○	○	○
Improved recognition of community pharmacy skills by patients	○	○	○	○	○
Other (please state) ______	○	○	○	○	○

Community of Practice

Where is your current region of pharmacy practice?

[▾]

How long have you been a UK (or NI) registered pharmacist or pharmacy technician?

[▾]

How did you hear about this survey?

Would you be interested in joining a community of practice for antimicrobial stewardship?

- Yes
- No

Please provide your details below so that we have permission to contact you regarding the draw for a £25 Amazon voucher and/or community of practice. Your details will not be linked to your survey responses

Name

Email address

Powered by Qualtrics
